# Aqueous Extract of *Garcinia Indica* Choisy Restores Glutathione in Type 2 Diabetic Rats

**DOI:** 10.4103/0975-1483.66806

**Published:** 2010

**Authors:** H Kirana, BP Srinivasan

**Affiliations:** *T.V.M. College of Pharmacy, Gandhinagar, Bellary-583103, Karnataka, India*; 1 *Department of Pharmacology, Delhi Institute of Pharmaceutical Sciences and Research (DIPSAR), M.B. Road, Sector-III, Pushp Vihar, New Delhi-110 017, India*

**Keywords:** *Garcinia indica*, glutathione, oxidative stress, type 2 diabetes

## Abstract

Significant depletion of glutathione (GSH-reduced form) was observed in type 2 diabetes due to oxidative stress. Hence the present study was aimed to investigate a drug which restores GSH along with its anti-diabetic activity. Aqueous extract of *Garcinia indica* at a dose of 100 mg/kg and 200 mg/kg was given orally to streptozotocin-induced type 2 diabetic rats for a period of 4 weeks. At the end, parameters such as fasting blood glucose, postprandial blood glucose, and GSH in blood were analyzed. Aqueous extract of *G. indica* significantly decreased both the fasting and postprandial blood glucose in type 2 diabetic rats. The extract also restored the erythrocyte GSH in type 2 diabetic rats. Drug at higher dose, i.e. 200 mg/kg, had a more pronounced effect. Restoring the erythrocyte GSH, an intracellular anti-oxidant in diabetes, will be beneficial specially by preventing the risk of developing complications.

## INTRODUCTION

Oxidative stress is the major cause and consequence of type 2 diabetes.[1] Glutathione (GSH), the most abundant thiol exists as GSH (reduced form) and GSSG (oxidized form) in cells, plays an important role against oxidative stress.[2] Depletion of intracellular GSH leads to neurodegeneration, myocardial infraction and other cardiovascular complications in diabetes.[3]

*Garcinia indica* Choisy. (Family: Guttiferae) is an evergreen tree found in the tropical rain forests of Western Ghats, from Konkan southwards in Mysore, Coorg, and Wynaad of India. The tree bears globose to spherical fruits of 4-5 cm in diameter during March-April. The fruit appears as dark purple when ripe enclosing 5-8 large seeds. Ripened fruit has agreeable flavor and sweetish acid taste.[4] The literature revealed the presence of polyisoprenylated benzophenone derivatives, namely garcinol, isogarcinol,[5] and organic acids chiefly contain (-)-hydroxycitric acid,[6] in the fruits. A small amount of xanthones and xanthone derivatives were also reported.[6] The dried rind of *G. indica* (Kokum) is traditionally used as a garnish for curry. In Ayurveda, the drug has anthelmintic and cardiotonic properties.Decoction of the fruit is used in treatment of diabetes.[7] Studies have shown the anti-oxidant and anti-glycation activities of *G. indica in vitro*.[8,9] In this view, we studied the effect of aqueous extract of *G. indica* on GSH in type 2 diabetic rats.

## MATERIALS AND METHODS

### Collection and authentication of plant drug

Fruits of *G. indica* Choisy. were collected from Udupi located in Karnataka, India. Drug was dried under shade at temperature not exceeding 40°C and authenticated (Accession No: Fru./B.2/R-3/sp-11) at Department of Raw Materials Herbarium and Museum, National Institute of Science Communication and Information Resources (NISCAIR), New Delhi.

### Aqueous extract and phyto-constituents

Dried fruit was grounded into a moderately coarse powder (# 22) in a domestic electric grinder. One part of the powdered drug was boiled with 16 parts of water for a period of 15 min and filtered through muslin cloth.[7] Filtrate was then evaporated under reduced pressure in Rota-rod evaporator (Buchi RE 121, Japan). The dried aqueous extract (22.7%) was packed in air tight container and stored in a desiccator for further studies. Phytochemical analysis[10] of the aqueous extract showed the presence of carbohydrates, xanthones as C-glycosides, tannins, and citric acid.

### Dose and drug solution—

In Ayurveda, 5 g of the powdered drug in the form of decoction is used in treatment of diabetes.[7] In considering the extractive value (22.7%) and rat metabolic rate (6 to 7 times higher than humans), we selected 100 mg/kg/day and 200 mg/kg/day doses of aqueous extract for screening the activity. The drug solution was prepared by dissolving the required quantity of aqueous extract in distilled water prior to the administration.

### Animals

*Wistar* albino rats (140-160 g) of either sex were housed under standard laboratory conditions at temperature 25 ± 2°C and 55 ± 5 % relative humidity with a regular 12 h light: 12 h dark cycle. Animals were given standard rat pellet and tap water *ad libitum*. The study protocol (Protocol number: IAEC/ 2004/ 06) was approved by Institutional Animal Ethical Committee (IAEC).

### Streptozotocin induced neonatal rat model for type 2 diabetes

Type 2 diabetes was induced by administering streptozotocin (90 mg/kg i.p.) in two-day-old neonatal rats. After 6 weeks of streptozotocin injection, rats showing the fasting blood glucose more than 160 mg/dl were considered as type 2 diabetes positive.[11,12]

### Experimental groups

Wistar albino rats of either sex were randomly allotted into five groups of six animals (n=6) each. Equal number of males and females were maintained in each group and caged separately. Group I served as normal and received distilled water. Group II served as type 2 diabetic control and received distilled water. Group III was type 2 diabetic treated with 100 mg/kg of aqueous extract of *G. indica*. Group IV was type 2 diabetic treated with 200 mg/kg of aqueous extract of *G. indica*.[7] Group V was type 2 diabetic treated with 10 mg/kg of gliclazide.[13] Drug treatment was done on every day morning with the help of oral catheter for a period of 4 weeks.[14] Body weight was determined at the end of every week. After 4 weeks, the parameters such as fasting blood glucose, postprandial blood glucose, and GSH in blood were analyzed.

### Estimation of fasting blood glucose

Blood samples were withdrawn from overnight fasted animals by retro-orbital puncture and centrifuged at 3000 rpm for 15 min, at 4°C in cooling centrifuge (Remi, C-24 BL, Mumbai, India). Glucose in serum was estimated by the glucose oxidase and peroxidase (GOD-POD kit) method.[15]

### Estimation of postprandial blood glucose

Overnight fasted animals were treated with respective drug solutions. Thirty minutes after the drug treatment, glucose solution at a dose of 2.5 g/kg body weight was administered orally with the help of a oral catheter. Blood samples were withdrawn after 120 min of oral glucose load (postprandial) and estimated the serum glucose by the glucose oxidase and peroxidase (GOD-POD kit) method.[16]

### Estimation of GSH in blood

0.2 ml of blood withdrawn from overnight fasted animals was added into 1.8 ml of distilled water. 3.0 ml of precipitating solution [1.67 g of glacial metaphosphoric acid, 0.2 g of disodium ethylene-diamine tetra-acetic acid (EDTA) and 30 g of sodium chloride dissolved in 100 ml distilled water] was added to the hemolysate and filtered. To 0.2 ml of the filtrate, 0.8 ml of phosphate solution (0.3 M Na_2_HPO_4_) and 0.1 ml of DTNB [5, 5’ dithiobis-2-nitrobenzoic acid] reagent were added. A stable yellow color formed was measured colorimetrically at 412 nm.[17] GSH in a sample was analyzed by using standard curve.

### Statistical analysis

Data are expressed as mean ± SEM. Statistical comparison between different groups was done using One-way analysis of variance (ANOVA) followed by the Tukey-Kramer multiple comparison test. *P*<0.05 was considered as statistically significant.

## RESULTS

### Effect on body weight

**Figure 1:**
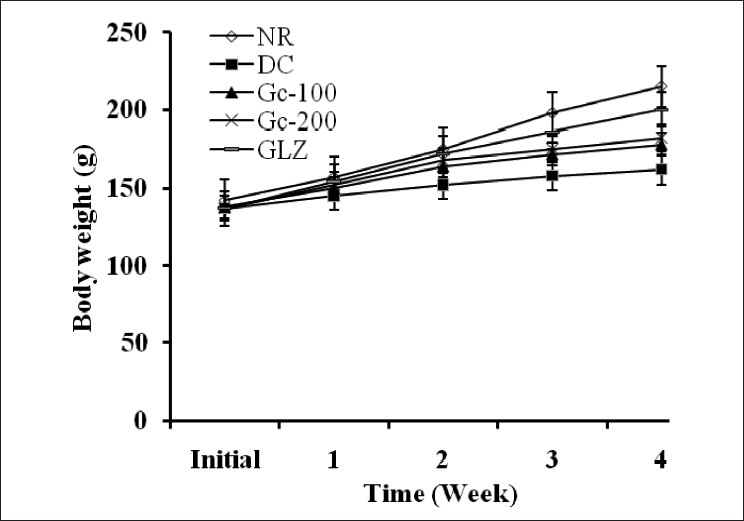
Effect of aqueous extract of *Garcinia indica* on body weight of type 2 diabetic rats [NR-Normal; DC = Type 2 diabetic control; Gc100-Type 2 diabetic treated with 100 mg/kg of aqueous extract of *G. indica*; Gc200-type 2 diabetic treated with 200 mg/kg of aqueous extract of *G. indica*; GLZ-type 2 diabetic treated with 10 mg/kg of gliclazide]. Values are expressed as mean ± SEM.

Two weeks of drug treatment significantly (*P*<0.05) improved the body weight of diabetic rats. Progress in weight gain of drug treated rats was continued for 4 weeks. By the end of fourth week, 100 mg/kg and 200 mg/kg dose of aqueous extract of *G. indica* showed 30.2% and 37.7% of relative weight gain respectively as compared to the diabetic control group. Gliclazide showed 72% gain in body weight. Body weight of various experimental groups initially i.e. before drug treatment and at the end of first, second, third, and fourth weeks of drug treatment is shown in Figure 1.

### Effect on fasting and postprandial blood glucose

Aqueous extract of *G. indica* at both doses, i.e. 100 mg/kg and 200 mg/kg, showed a significant (*P*<0.001) decrease in fasting blood glucose of type 2 diabetic rats. Difference in-between 100 mg/kg and 200 mg/kg dose was found to be significant (*P*<0.05) when analyzed for inter-group comparison, indicating that the drug at higher dose will be better effective. Elevated postprandial blood glucose of type 2 diabetic rats was significantly (*P*<0.001) decreased with the drug treatment [Table 1]. However, the aqueous extract of *G. indica* at both doses was unable to maintain the postprandial blood glucose below 140 mg/dl (normal).

**Table 1:** Effect of aqueous extract of *Garcinia indica* on blood glucose and GSH in type 2 diabetic rats

Group (n=6)	Fasting blood glucose (mg/dl)	Postprandial blood glucose (mg/dl)	GSH (μ moles/g Hb)
Normal	87.05 ± 2.71	114.82± 3.3	6.54 ± 0.12
Type 2 diabetic control	189.78 ± 3.64	305.33± 8.02	4.17 ± 0.12
Type 2 diabetic treated with 100 mg/kg of *G. indica*	142.16 ± 3.36**	185.33± 4.26**	4.95 ± 0.42*
Type 2 diabetic treated with 200 mg/kg of *G. indica*	127.33 ± 3.42**	157.05 ± 3.81**	5.58 ± 0.15**
Type 2 diabetic treated with 10 mg/kg of gliclazide	105.54 ± 2.35**	132.66 ± 3.74**	

Values are expressed as mean ± SEM;

* *P*<0.01,

** *P*<0.001, significantly different as compared to diabetic control

### Effect on GSH

Aqueous extract of *G. indica* with 100 mg/kg dose increased the erythrocyte GSH at *P*<0.01 level of significance and 200 mg/kg dose increased the erythrocyte GSH at *P*<0.001 level of significance. Higher dose of the extract had a more pronounced effect on restoring the erythrocyte GSH in type 2 diabetic rats. Statistical data of GSH in various experimental groups is shown in Table 1. GSH is expressed as μ moles to that of hemoglobin (g) measured in each blood sample.

## DISCUSSION

Most of the currently available anti-diabetic therapies reduce the fasting blood glucose but have a little impact on postprandial hyperglycemia. Postprandial hyperglycemia is an independent risk factor for cardiovascular diseases.[18] Aqueous extract of *G. indica* had a significant (*P*<0.001) effect on both fasting and postprandial hyperglycemia of type 2 diabetic rats. Although unable to maintain the normal levels in case of postprandial hyperglycemia, i.e. 140 mg/dl, the extract had a marked effect on elevated glucose levels. The extract at higher dose has more pronounced effect on type 2 diabetes. Body weight of type 2 diabetic rats was found to be less during the course of development as compared to normal animals. Weight loss in diabetes is generally due to continuous excretion of glucose from the body.[19] Improved body weight of the treated animals indicates the efficacy of *G. indica* in controlling the glucose excretion and blood glucose level of type 2 diabetic rats. In hyperglycemia, auto-oxidation of glucose increases the formation of free radicals beyond the capacity of defense system to neutralize it and cause oxidative stress.[1] One of the major etiologies in pathogenesis of diabetic complications is oxidative stress. Complications account for disabilities and high mortality rates in diabetes.[3] Aerobic cells are endowed with extensive antioxidant defense mechanisms including both low molecular weight scavengers such as reduced glutathione (GSH), ascorbic acid (vitamin C), Vitamin E and enzyme system such as superoxide dismutase (SOD), catalase (CAT) and glutathione peroxidase (GSH-Px).[2] A non-enzymatic endogenous antioxidant GSH (reduced form) plays an important role against oxidative stress in diabetes. Significant (*P*<0.001) depletion of GSH was observed in type 2 diabetic rats as compared to normal group due to oxidative stress. Treatment with the aqueous extract of *G. indica* enabled the rats to restore GSH. The drug at 200 mg/kg dose has better effect on restoring the GSH in type 2 diabetic rats. Aqueous extract of *G. indica* contain 12-15% of total organic acids of which chief constituent is the (-)-hydroxycitric acid.[4,20] These organic acids which act as strong antioxidants may be responsible for preventing the depletion of GSH under stress conditions. Anti-oxidant potential of garcinol[8,9] present in *G. indica* may also be responsible for GSH restoration in type 2 diabetic rats. Isolated (-)-hydroxycitric acid has been reported to posses regulatory effect on carbohydrate and lipid metabolism.[20] It limits the availability of acetyl-CoA required for fatty acid synthesis and promotes glycogenesis, β-oxidation, etc. These metabolic pathways initiate the uptake of glucose by the muscle and thereby combat insulin resistance of type 2 diabetes.[14] Marked reduction in the fasting and postprandial hyperglycemia by the aqueous extract of *G. indica* may be related to the above regulatory effects of drug on metabolism. Further studies on *G. indica* in diabetes-related complications will assure the potential benefit of multidimensional activity of the plant drug in type 2 diabetes.

## CONCLUSION

*G. indica* an Ayurvedic drug having anti-diabetic activity along with GSH restoration is beneficial in treatment of type 2 diabetes specially by preventing the risk of developing complications.
